# Development and psychometric properties of the Portuguese version of the Orofacial Esthetic Scale: OES-Pt

**DOI:** 10.7717/peerj.8814

**Published:** 2020-03-17

**Authors:** Lucas A. Campos, João Marôco, Mike T. John, Ary Santos-Pinto, Juliana A.D.B. Campos

**Affiliations:** 1 Department of Pediatric Dentistry and Orthodontics/School of Dentistry, São Paulo State University, Araraquara, São Paulo, Brazil; 2 William James Center for Research (WJCR), University Institute of Psychological, Social, and Life Sciences (ISPA), Lisbon, Portugal; 3 Department of Diagnostic and Biological Sciences/School of Dentistry, University of Minnesota, Minneapolis, MN, USA; 4 Department of Food Science and Nutrition/School of Pharmaceutical Sciences, São Paulo State University, Araraquara, São Paulo, Brazil

**Keywords:** Psychometrics, Dental esthetics, Validation studies, Structural equation model

## Abstract

**Background:**

The Orofacial Esthetic Scale (OES) is an instrument to assess an individual’s perception of their Orofacial Appearance (OA). However, its translation and evaluation of psychometric properties is necessary for its use in Brazilian individuals.

**Objectives:**

To develop the Portuguese version of OES (OES-Pt), estimate its psychometric properties (validity, measurement invariance and reliability) when applied to Brazilian individuals aged 18–40 years, and estimate the relationship between sociodemographic characteristics and OA.

**Methods:**

This was a cross‐sectional study using a convenience sample. The sample consisted of 1,072 Brazilian individuals (70.1% female, 25.1% dental patients; mean ± SD age: 25.7 ± 5.7 years). After cross-cultural adaptation of OES-Pt, factorial validity was evaluated by confirmatory factor analysis. Convergent validity (average variance extracted (AVE)) and reliability (Cronbach’s alpha coefficient (α) and Composite Reliability (CR)) were also estimated. Concurrent validity was assessed (Pearson’s correlational analysis (*r*) between OES-Pt total score and item eight of the OES which refers to global assessment of OA). Measurement invariance of the factorial model (multigroup analysis using ΔCFI) was evaluated for independent samples (sample randomly split into two: “Test Sample” and “Validation Sample” and according to sex: male and female, age range: 18–30 and 31–40 years, and whether the individual is undergoing dental treatment or not). A Structural Equation Model estimated the relationship between sociodemographic characteristics and OA.

**Results:**

OES-Pt presented adequate fit to the sample. Convergent validity (AVE ≥ 0.56) and reliability (α and CR ≥ 0.89) were adequate. Concurrent validity was adequate (*r* = 0.88; *p*-value < 0.001). OES-Pt presented strict invariance for independent samples. Age, sex, and socioeconomic status (SES) were related to OA, indicated by standardized beta coefficients (standardized β) of 0.036 (standard error: 0.007), 0.001 (0.094) and 0.196 (0.061), respectively on OA. These three relationships were either weak or not statistically significant.

**Conclusions:**

When measuring OA in Brazilian individuals, the OES-Pt was valid, reliable and invariant for independent samples. Age, sex and SES were weak or not statistically significantly related to OA.

## Introduction

Orofacial Appearance (OA) is one of the four dimensions of oral health-related quality of life (OHRQoL) (John et al., 2014a, 2014b). It substantially affects an individual’s social and subjective well-being (Ament & Ament, 1970; Klima, Wittemann & McIver, 1979). Consequently, OA is one of the most important dental patient-reported outcomes (John, 2018). The evaluation of OA is relevant for clinical decision-making and treatment outcomes (Hua, 2019; Reissmann, 2019).

As the perception of OA is subjective, it differs between a dentist and a dental patient (Hua, 2019) and the perspective of the latter should prevail for setting expectations regarding the need or demand for as well as outcome of treatment. Perceptions about OA could guide decision-making of clinical management in two aspects. First, it quantifies demand for treatment from the patient’s point of view. Second, it contributes to a clinical treatment plan that meets these demands and the patient’s expectations. This assessment may be the starting point for conducting a process that achieves patient satisfaction with treatment.

Orofacial Appearance and the other dimensions of OHRQoL cannot be directly observed, hence, questionnaires are typically used for assessing these concepts. These questionnaires, when psychometrically sound, allow standardization of measures resulting in a compatibility of the results with other patients or with subsequent assessments of the same patient during treatment. Examples of instruments that can be used to evaluate general OHRQoL are Geriatric Oral Health Assessment Index (Atchison & Dolan, 1990), Oral Impacts on Daily Performances (Adulyanon & Sheiham, 1997) and Oral Health Impact Profile (Slade & Spencer, 1994). However, these instruments have few items with content related to esthetic aspects, not being sufficient to specifically evaluate the perception of OA (Larsson et al., 2010; Mehl et al., 2009). Thus, to assess the direct aspects of OA, it is necessary to use a specific instrument.

An instrument for evaluating OA is the Orofacial Esthetic Scale (OES). This scale was originally developed in Sweden, along with an English version, to evaluate OA in prosthodontic patients (Larsson et al., 2010). The OES has one total score (Larsson et al., 2010). The instrument has already been translated and adapted for different countries (Alhajj et al., 2017; Bimbashi et al., 2015; N’Guyen-Van, Moreau & Braud, 2019; Persic et al., 2011; Reissmann et al., 2015; Simancas-Pallares et al., 2018; Wetselaar et al., 2015; Zhao & He, 2013) and applied to different contexts, such as the general population (John et al., 2012), specific dental patients such as denture wearers (Larsson et al., 2010), and dental patients in general (Reissmann et al., 2019). In addition, some studies have evaluated the psychometric properties of OES, attesting to its validity and reliability for the Swedish general population (John et al., 2012) and for the population of North American dental patients (Reissmann et al., 2019). For Portuguese, one of the world’s most widely spoken languages, an OES version for use in Lusophone individuals does not exist.

The relationship between sex and age, on one hand and OA, on the other hand, has been investigated similarly to studies investigating such relationships for the construct OHRQoL (Pakpour et al., 2018); however, there is limited knowledge whether this relationship is clinically relevant. The literature indicates the level of OA impairment is different between sexes, with women having greater psychosocial impact and dissatisfaction with OA (Kang & Kang, 2014; Reissmann et al., 2019). Regarding age, older individuals are less satisfied with OA (Carlsson et al., 2014).

In addition, dental patients’ perception of their orofacial esthetics varies from individuals not undergoing treatment, since he/she has greater attention paid to the orofacial region compared to individuals who are not being treated (Zucoloto, Maroco & Campos, 2016). Therefore, understanding the differences in OA among individuals with different characteristics is important for identifying the demand for esthetic dental treatment and for clinical decision making.

The aims of the present study were to: (i) develop the Portuguese version of the OES (OES-Pt); (ii) estimate its psychometric properties and measurement invariance in subsamples of different characteristics (sex, age and dental treatment or not); and (iii) determine the relationship between sociodemographic characteristics and OA.

## Methods

### Study design and sampling

This was a cross-sectional study with a convenience sample. Initially, dental patients (in the clinic waiting room) and employees of São Paulo State University were invited to participate in the study. Then, a snowball strategy was used to recruit more participants. Individuals included in the study were male and female (biological status) adults between 18 and 40 years of age. The individuals who did not identify with their biological sex were instructed not to answer this question in the demographic questionnaire and subsequently they were excluded from the study. The age range was limited to 40 years since the perception of body image satisfaction may change from young to mature adulthood (Cash & Smolak, 2011). This can bias the perception of OA satisfaction by physical changes in facial characteristics such as the appearance of wrinkles on the forehead and around the eyes (Cash & Smolak, 2011; Persic et al., 2011).

While some authors suggest a fixed number of participants for structural equation models (SEM), for example, more than 200 participants (Chang et al., 2018; Su et al., 2014), other authors emphasize that several factors affect sample size requirements (Kyriazos, 2018). For the present study, the proposal of Hair et al. (2009) was used for the calculation of the minimum sample size. They recommended a minimum of 5–10 participants per model parameter. Considering that the factorial model to be tested has 13 parameters, the minimum sample size was 65–130 individuals. However, because we aimed to determine psychometric properties of OES for independent samples (“Test Sample” and “Validation Sample”) and for the population with different sample characteristics (such as sex: male and female, age range: 18–30 and 31–40 years, and whether the individual is undergoing dental treatment or not) the sample should be large enough in each category of each of these characteristics. Therefore, we recruited a larger number of study participants.

### Study variables

Information such as sex (biological status: male and female), age, marital status (single, married, divorced and widower), socioeconomic status (SES), if the individual is undergoing or has had any dental treatment with the main objective of improving dental esthetics (no/yes), if the individual likes her/his own smile (no/yes), and if the individual is currently undergoing any type of dental treatment (no/yes) were collected for sample characterization. The SES was estimated according to the Brazilian Criteria (Brazilian Market Research Association (ABEP), 2018). The individuals were classified into socioeconomic stratum D–E (mean monthly income: R$ 708.19, U$ 175.63); C (R$ 1,691.69–2,965.69; U$ 419.54–735.50); B (R$ 5,363.19–10,386.52, U$ 1,330.09–2,575.89); and A (R$ 23,345.11, U$ 5,789.67).^1^ 1The values were estimated from the Central Bank of Brazil quotation on May 27, 2019–U$ 1.00 = R$ 4.03.

### Measurement of OA

The OES is a unidimensional instrument to measure OA. It contains seven items to assess specific esthetic aspects (face, facial profile, mouth, gum and alignment, shape and color of teeth). Also, the instrument has one item for global assessment of OA, which is not considered as a component of the factorial model. The response scale is a 11-point numerical rating scale ranging from 0 (very dissatisfied) to 10 (very satisfied) (Larsson et al., 2010).

### Development of the OES-Pt

For the development of the OES-Pt, three translators (native speakers of Portuguese with English proficiency) independently translated OES from English to Portuguese. The translations were compared by the researchers to create a single Portuguese version of the instrument (Beaton et al., 2000).

The conceptual and cultural equivalences of the Portuguese version were evaluated by two researchers with knowledge in dentistry and psychometrics. It was verified that the items’ content of the translated version was in agreement with the theoretical construction proposed by the original authors (Larsson et al., 2010) and also with the Brazilian context. After the Portuguese version was established, this version was analyzed by one Portuguese researcher aiming to obtain a Portuguese version reconciled according to the orthographic agreement established between Portuguese-speaking countries in 2009 (http://www.portaldalinguaportuguesa.org/acordo.php?action=acordo&version=1990).

This reconciliation was made to produce a version that can be used more broadly among different Lusophony contexts. Then, the suggestions were analyzed by the researchers of the present study and the intermediate version in reconciled Portuguese was obtained. This intermediate version was tested in a pilot study.

### Psychometric properties analysis

#### Item characteristics

The distribution of the responses given to the OES-Pt items was determined using measures of central tendency, variability, and shape of the distribution. Absolute values of skewness and kurtosis below 3 and 10, respectively, were indicative of approximation to the normal distribution (Kline, 2016). Multivariate normality was evaluated using Mardia’s Test, values lower than 3 were considered indicative of multivariate normality.

#### Construct validity

Construct validity of OES-Pt was assessed using factorial and convergent validity. The factorial model tested was composed of seven items that evaluated the specific esthetic aspects (it1–it7), as in the original instrument (Larsson et al., 2010). Factorial analysis was estimated using Confirmatory Factor Analysis with the Maximum Likelihood Estimation method. The following parameters were used to evaluate goodness-of-fit of the model to the data: the ratio of chi-square to degrees of freedom (χ^2^/df), comparative fit index (CFI), Tucker–Lewis index (TLI), root mean square error of approximation (RMSEA) and standardized root mean square residual (SRMR) (Hu & Bentler, 1999; Marôco, 2014). The factor loadings of the items (λ) were also considered. The fit of the model was considered adequate when χ^2^/df ≤ 2,0; CFI and TLI > 0.90, RMSEA < 0.10, SRMR < 0.08 and λ ≥ 0.50 (Hu & Bentler, 1999; Marôco, 2014). Modification indices estimated from the Lagrange Multiplier (LM) were inspected (LM > 11) to verify existence of correlation between item errors (Marôco, 2014).

Considering the need to evaluate the model fit in independent samples to assess the external validity of the results, the total sample was randomly divided into two parts named “Test Sample” (*n* = 535) and “Validation Sample” (*n* = 537). The invariance was estimated by multigroup analysis using the CFI difference (ΔCFI) for factor loadings (λ), intercepts (i) and residuals (Res). Invariance was assumed when the absolute value of ΔCFI was less than 0.01. Weak invariance was considered when the factor loadings of the models do not differ significantly (metric invariance). If invariance was observed in the factor loadings and intercept (scalar invariance), strong invariance was considered. Strict invariance was considered if no significant difference in factor loadings, intercept and residuals is observed.

To verify invariance of the model according to the characteristics of interest, the sample was subdivided according to sex (male vs female), age (18–30 vs 31–40 years) and whether the individual is undergoing dental treatment (individuals currently under dental treatment vs individuals not currently under dental treatment). To perform the invariance testing, samples with similar sizes were randomly selected due to the sample size discrepancy between the categories of the variables (sex: male: *n* = 314, female: *n* = 336; age: 18–30 years: *n* = 270, 31–40 years: *n* = 260; individuals currently under dental treatment: *n* = 269; individuals not currently under dental treatment: *n* = 268). The invariance of the factorial model between the subsamples was estimated as described above.

Convergent validity was estimated from Average Variance Extracted (AVE) following Fornell and Larcker’s proposal (Fornell & Larcker, 1981). A value of AVE ≥ 0.50 was considered adequate.

#### Concurrent validity

The concurrent validity of OES-Pt was assessed using Pearson’s correlational analysis (*r*) between OES and item eight, which refers to global assessment of OA. The linearity was assessed using the standardized residual covariance matrix (OES8 vs all OES-Pt items = 0.03–1.44 in absolute values).

#### Reliability

Reliability was estimated using the standardized Cronbach’s alpha coefficient (α) and Composite Reliability (CR) (Fornell & Larcker, 1981). CR and α ≥ 0.7 were considered adequate.

#### Criterion for discriminant validity

To verify whether OES discriminates between individuals currently under dental treatment and individuals not currently under treatment, first the invariance of the factorial model for these subsamples was verified. If invariance was observed, the mean OES-Pt score (calculated for each individual from using the mean of the answers given to the seven component items of the factorial model) was compared between these groups.

Due to the large sample size, normality was assessed from the shape of the distribution measurements. Absolute values of skewness and kurtosis below 3 and 10, respectively, were indicative of approximation to the normal distribution (Kline, 2016). The homoscedasticity of the data was assessed using Levene’s test. Data presented approximations to the normal distribution (skewness ≤ |0.8| and kurtosis ≤ |0.76|). Data heteroscedasticity was observed (Levene’s test: *F* = 13.71, *p* < 0.001). The comparisons were performed using Welch’s *t*-test (for unequal variances). The significance level adopted was 5% (two-sided).

### Structural equation model

A SEM estimated the relationships between sociodemographic characteristics (independent variables: sex (binary category), age (in years) and SES (in ordinal category)) and OA (dependent variable). The fit of the model was assessed using goodness of fit indices mentioned in the “Construct validity”. The standardized beta coefficients (standardized β) used to indicate the relationships between independent variables and OA were estimated and evaluated using the two-tailed *z* test (α = 5%). The scores used for the categorical variables of the model were: sex (0 = male and 1 = female); SES^2^ 2Brazilian Economic Classification Criteria (Brazilian Market Research Association (ABEP), 2018), mean income per stratum: D-E = R$ 708.19, U$ 175.63; C = R$ 1,691.69–2,965.69; U$ 419.54–735.50; B = R$ 5,363.19–10,386.52, U$ 1,330.09–2,575.89; A = R$ 23,345.11, U$ 5,789.67. Estimated from the quotation of 05/27/2019 of the Central Bank of Brazil (U$1.00 = R$4.03). (1 = D/E, 2 = C, 3 = B and 4 = A). Values of standardized β < 0.2 are considered weak, 0.2 ≤ standardized β < 0.5 moderate and standardized β ≥ 0.5 strong relationship (Acock, 2014).

The analyses were performed in IBM SPSS Statistics 22 (IBM Corp., Armonk, NY, USA) and AMOS 22.0 (IBM Corp., Armonk, NY, USA) software.

### Ethical approval

Only individuals who agreed and signed the written Informed Consent participated in the study. The study was approved by the Research Ethics Committee of São Paulo State University (Unesp), School of Dentistry, Araraquara (CAAE: 88600318.3.0000.5416).

## Results

### Pilot study

Fifty-eight individuals participated in this study (81% women; mean age 28.4 (standard deviation = 5.5) years; 72.4% single, 24.1% married, 3.5% divorced; 6.9% are in dental treatment to improve esthetics and 60.3% have already had dental treatment for this purpose; 81.0% of participants like their own smile; 1.7% economic stratum D/E, 20.7% economic stratum C, 65.5% economic stratum B and 12.1% economic stratum A; and 20.7% are dental patients). The median of the time to complete OES-Pt was 56 s (minimum value = 18 s, first quartile = 46.5 s, third quartile = 73 s, maximum value = 149 s). The Incomprehension Index (II) was estimated to identify possible difficulties in understanding the items’ content by participants (Campos et al., 2019). II > 15% was considered indicative of the need for reformulation of the item (Campos et al., 2019). All OES-Pt items presented II < 2%, and therefore, the understanding of the items was considered adequate. Table 1 presents the final OES-Pt.

**Table 1:** Orofacial Esthetic Scale.

	English version^†^	Portuguese version (OES-Pt)
Instruction	How do you feel about the appearance of your face, mouth, teeth, and your tooth replacements (crowns, bridges, and implants)? 0 = very dissatisfied, 10 = very satisfied	Como você se sente em relação à aparência dos seus dentes, boca e face (rosto). 0 = muito insatisfeito (a), 10= muito satisfeito (a)
Item		
it1	Your facial appearance	Sua aparência facial
it2	Appearance of your facial profile	Aparência de seu perfil facial
it3	Your mouth’s appearance (smile, lips, and visible teeth)	Aparência de sua boca (sorriso, lábios e dentes visíveis)
it4	Appearance of your rows of teeth	Aparência do alinhamento dos seus dentes
it5	Shape/form of your teeth	Formato de seus dentes
it6	Color of your teeth	Cor de seus dentes
it7	Your gum’s appearance	Aparência de sua gengiva
it8	Overall, how do you feel about the appearance of your face, your mouth, and your teeth?	No geral, como você se sente em relação à aparência de sua face, boca e dentes?

**Note:**

† Larsson et al., 2010.

### Psychometric properties analysis

A total of 1,135 individuals participated in the study. Of these participants, 63 individuals were excluded because they did not answer all OES-Pt items. The mean age was 25.7 years (standard deviation = 5.7 years). Table 2 shows the sample characterization. Most of the participants were women, single, had been in some kind of esthetic dental treatment, liked their own smile, and were not undergoing dental treatment at the time of participation. Splitting the sample into two sets (Test Sample and Validation Sample) showed no substantial difference in these characteristics.

**Table 2:** Participants characteristics (mean ± standard deviation or n (%)).

Characteristic	Sample					
	Total sample (*n* = 1,072)		Test sample (*n* = 535)		Validation sample (*n* = 537)	
Age (years)	25.7 ± 5.7		26.1 ± 5.8		25.4 ± 5.6	
Sex						
Female	758 (70.7)		386 (72.1)		372 (69.3)	
Marital status						
Single	832 (77.9)		406 (76.4)		426 (79.5)	
Married	213 (19.9)		113 (21.2)		100 (18.6)	
Divorced	22 (2.1)		13 (2.4)		9 (1.7)	
Widower	1 (0.1)		–		1 (0.2)	
Economic stratum^†^						
A	269 (25.1)		144 (26.9)		125 (23.3)	
B	565 (52.8)		274 (51.2)		291 (54.3)	
C	225 (21.0)		107 (20.0)		118 (22.0)	
D/E	12 (1.1)		10 (1.9)		2 (0.4)	
Are you undergoing dental treatment?						
Yes	269 (25.1)		123 (23.0)		146 (27.2)	
Are you currently under esthetic dental treatment?						
Yes	79 (7.4)		25 (4.7)		54 (10.1)	
Have you ever been under any kind of esthetic dental treatment in your life?						
Yes	644 (60.7)		325 (61.2)		319 (60.2)	
Do you like your smile?						
Yes	837 (79.3)		415 (79.2)		422 (79.3)	

**Note:**

† Brazilian Economic Classification Criteria (Brazilian Market Research Association (ABEP), 2018), mean income per stratum: D–E, R$ 708.19, U$ 175.63; C, R$ 1,691.69–2,965.69, U$ 419.54–735.50; B, R$ 5,363.19–10,386.52, U$ 1,330.09–2,575.89; A, R$ 23,345.11, U$ 5,789.67. Estimated from the quotation of 05/27/2019 of the Central Bank of Brazil (U$1.00 = R$4.03).

Table 3 presents the summary measures of the responses given to OES items according to the Sample (Test or Validation). All items presented approximation to the normal distribution. In addition, the data showed no evidence against multivariate normality (Mardia’s Test: 1.458).

**Table 3:** Descriptive statistics of the responses given by the participants of the test (*n* = 535) and validation (*n* = 537) samples to the items of the Portuguese version of Orofacial Esthetic Scale (OES-Pt).

Item	Test sample/validation sample				
	Mean	Median	Standard deviation	Skewness	Kurtosis
It1	7.1/7.0	7/7	2.0/2.0	−0.8/−1.0	1.1/1.3
It2	6.8/6.7	7/7	2.2/2.4	−0.7/−0.7	0.4/0.2
It3	7.1/7.0	8/8	2.4/2.4	−0.9/−1.0	0.4/0.8
It4	6.9/6.7	8/7	2.7/2.7	−0.9/−0.8	0.0/0.0
It5	7.2/7.1	8/8	2.6/2.6	−1.0/−1.1	0.2/0.7
It6	6.3/6.1	7/7	2.7/2.7	−0.6/−0.6	-0.4/-0.4
It7	7.5/7.4	8/8	2.4/2.4	−1.0/−1.0	0.6/0.5
It8	7.4/7.2	8/8	2.1/2.1	−1.1/−1.2	1.5/1.7

**Figure 1:**
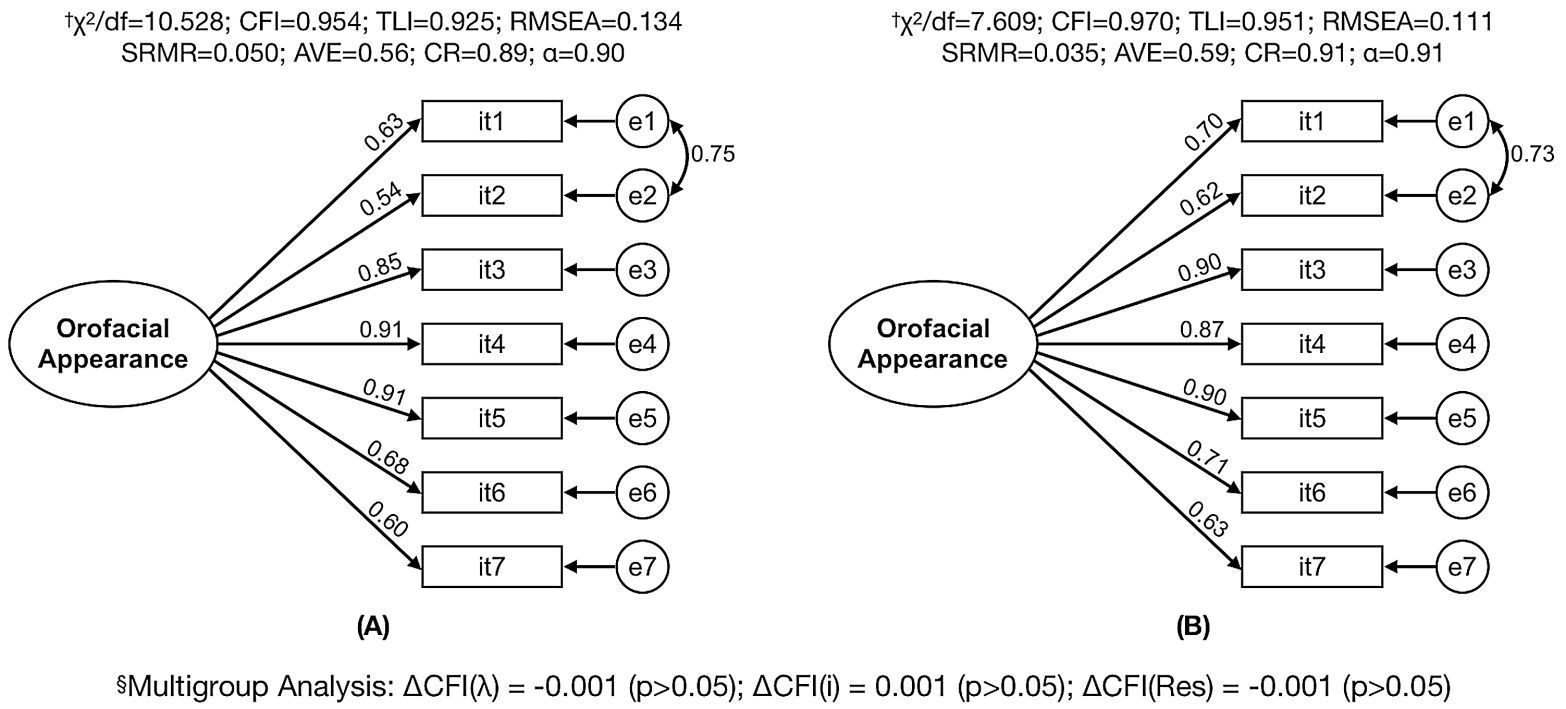
Factorial model fit of the Orofacial Esthetic Scale (OES-Pt) for test and sample data and multigroup analysis between samples. (A) Test sample (*n* = 535); (B) validation sample (*n* = 537); ^†^χ^2^/df, ratio of chi-square to degrees of freedom; CFI, comparative fit index; TLI, Tucker–Lewis index; RMSEA, root mean square error of approximation; SRMR, standardized root mean square residual; AVE, average variance extracted; CR, composite reliability; α, standardized Cronbach’s alpha coefficient; ^§^ΔCFI, comparative fit index difference; λ, factor loading; i, intercept; Res, residuals.

The factorial model of OES-Pt did not present adequate fit to the Test Sample data (λ = 0.60–0.89; χ^2^/df = 39.269; CFI = 0.800; TLI = 0.701; RMSEA = 0.268 and SRMR = 0.092). Inspecting the values of LM, correlation between the errors of items 1 (face) and 2 (facial profile) was observed (LM = 302.032). Therefore, the adequate fit of the model was obtained with the insertion of correlation between items 1 and 2. It should be noted that RMSEA did not reach the suggested threshold value. However, it is noteworthy that RMSEA is overestimated in simple factorial models (with few degrees of freedom), such as the OES factorial model (Kenny, Kaniskan & McCoach, 2015). Thus, the SRMR index (a fit index that does not include the chi-squared value) was considered as an alternative to the RMSEA in order to make a decision regarding adequate fit. The model presented adequate factorial and convergent validity and reliability for the “Test Sample” and “Validation Sample” data (Fig. 1). There is also a strict invariance between the samples, pointing to adequate external validity of the results (Fig. 1). Also, a high correlation was observed between the OES-Pt and the response given to item 8 of the OES-Pt (*r* = 0.88; *p*-value < 0.001), pointing to an adequate concurrent validity of this scale.

Table 4 presents the result of the multigroup analysis performed to evaluate the invariance of the model between the subsamples (male vs female; younger vs older; and individual currently under dental treatment vs individual not currently under dental treatment). The refined factorial model of OES-Pt presented adequate fit to all subsamples. In addition, a strong invariance (scalar) between sexes and whether the individual is undergoing dental treatment or not was observed. Strict invariance between the younger and older sample was observed.

**Table 4:** Goodness of fit indices of the factorial model of Orofacial Esthetic Scale (OES-Pt) applied to the different subsamples (Male, Female, Younger, Older, Individual currently under dental treatment, Individual not currently under dental treatment) and multigroup analysis to evaluate invariance of the model.

Subsample	CFA^†^							ΔCFI^‡^ (*p*)		
	*n*	χ^2^/df	CFI	TLI	RMSEA	SRMR	λ	λ	i	Res
Male	314	5.611	0.963	0.940	0.121	0.041	0.60–0.88			
Female	336	6.189	0.963	0.939	0.124	0.046	0.55–0.92			
Male vs Female	650	5.410	0.938	0.946	0.082	0.052	0.58–0.91	−0.002 (*p* > 0.05)	−0.005 (*p* > 0.05)	−0.018 (*p* < 0.05)
Younger^§^	270	4.397	0.965	0.943	0.112	0.044	0.55–0.88			
Older^¶^	260	4.890	0.969	0.950	0.123	0.040	0.64–0.95			
Younger vs older	530	3.788	0.954	0.960	0.073	0.058	0.60–0.91	−0.003 (*p* > 0.05)	−0.005 (*p* > 0.05)	−0.005 (*p* > 0.05)
Individual under dental treatment	269	7.087	0.947	0.914	0.151	0.054	0.61–0.89			
Individual not currently under dental treatment	268	5.452	0.960	0.936	0.129	0.040	0.58–0.91			
Under treatment vs not under treatment	537	4.856	0.937	0.945	0.085	0.055	0.62–0.90	−0.001 (*p* > 0.05)	−0.005 (*p* > 0.05)	−0.011 (*p* < 0.05)

**Notes:**

† CFA, confirmatory factor analysis; χ^2^/df, ratio of chi-square to degrees of freedom; CFI, comparative fit index; TLI, Tucker–Lewis index; RMSEA, root mean square error of approximation; SRMR, standardized root mean square residual; λ, factor loading.

‡ ΔCFI, comparative fit index difference; λ, factor loading; i, intercept; Res, residuals.

§ Younger sample: 18–30 years.

¶ Older samples: 31–40 years.

Regarding OES-Pt scores, individuals currently under dental treatment (*n* = 269; mean ± standard deviation: 6.4 ± 2.2; 95% CI [6.1–6.7]) had significantly lower scores than the individuals not currently under dental treatment (*n* = 803, mean ± standard deviation: 7.1 ± 1.8; 95% CI [7.0–7.2]) (Welch’s *t*-test: *t* = 4.75, *p* < 0.001; mean difference: 0.7, standard error: 0.15; 95% CI [0.4–1.0]).

### Structural equation model

In the structural model, only SES had a statistically significant relationship with OA (standardized β = 0.196, β = 0.369, standard error = 0.061, *p* < 0.001). However, it is noteworthy that this was a weak relationship. Also, it was observed that sex (standardized β = 0.001, β = 0.003, standard error = 0.094, *p* = 0.974) and age (standardized β = 0.036, β = 0.008, standard error = 0.007, *p* = 0.257) were not statistically significantly related to OA.

## Discussion

This study presented the OES-Pt and confirmed the validity and reliability of this instrument’s scores when applied to Brazilian adult individuals. Sociodemographic characteristics were either weak or not statistically significantly related to OA.

The psychometric properties of OES were evaluated after conceptual and cultural equivalence of the Portuguese version was established. It was necessary to allow for a correlation between the errors of items 1 (“facial appearance”) and 2 (“facial profile appearance”) to obtain an adequate fit of the factorial model to the data. Thus, unidimensionality of OES was confirmed, consistent with what was observed in other versions of this instrument (Bimbashi et al., 2015; John et al., 2012; Larsson et al., 2010; Reissmann et al., 2015, 2019; Zhao & He, 2013). Although John et al. (2012) pointed to a plausible theory that OA assessed by OES has two factors, the authors recommend that the one-dimensional proposal should be the choice because they report that the presence of two distinct aspects does not contribute to improving the estimate of the OA concept.

The measurement invariance of the OES-Pt factorial model in independent samples and according to sex and age, between the general population and dental patients indicates that this scale operates similarly in these subsamples to capture the concept of OA. Thus, although OES was originally proposed for prosthodontic patients (Larsson et al., 2010), the results of the present study corroborate earlier findings (John et al., 2012) allowing the use of this instrument in general population individuals.

The adequate validity (construct, convergent and concurrent) and reliability of OES-Pt observed in different subsamples strengthens the use of the instrument to obtain more accurate evidence related to OA in this population. The instrument has been successfully implemented in other cultures/countries as well (Bimbashi et al., 2015; John et al., 2012; Larsson et al., 2010; Reissmann et al., 2015, 2019; Zhao & He, 2013), indicating this is a robust instrument.

The results also corroborate previous studies (Bimbashi et al., 2015; Zhao & He, 2013), pointing out the ability of OES to discriminate between subjects currently under dental treatment and subjects not currently under treatment. This difference may be related to the fact that dental patients presented some dissatisfaction with aspects related to oral health (Zucoloto, Maroco & Campos, 2016). There was no significant difference between males and females with OA scores, indicating similar perception of orofacial components.

Individuals with higher SES showed greater OA compared to individuals with lower SES. This result may be related to the fact these individuals have more resources and better access to preventive interventions and better oral hygiene habits (Paula et al., 2012). Also, there was no significant difference in OA in relation to age, contrary to the findings of Carlsson et al. (2014), in which older individuals presented greater dissatisfaction with OA. This may have been due to the difference in the age range of Carlsson’s study (Carlsson et al., 2014) and ours. The present study investigated 18–40-year-olds whereas Carlsson’s upper age limit was 81 years.

The narrow age range of our study participants can be considered a limitation. Originally OES was studied in individuals with a wide age range (ranging from 22 to 82 years) (Larsson et al., 2010). Good psychometric properties have been reported in studies with a broad age range (Alhajj et al., 2017; Bimbashi et al., 2015; John et al., 2012; N’Guyen-Van, Moreau & Braud, 2019; Persic et al., 2011; Reissmann et al., 2015; Simancas-Pallares et al., 2018; Wetselaar et al., 2015; Zhao & He, 2013) and consequently, we do not see a reason not to generalize OES’ psychometric properties to the entire adult age range. Another limitation of the study is the convenience sample, in which participants were initially recruited at the São Paulo State University and then a snowball strategy was adopted, but limited to the southeastern region of the country. As culture and demographic characteristics may vary in different regions of the country, generalizing the results of the present study to the entire Brazilian population should be cautioned. However, this convenience sample strategy is commonly used in validation studies (Campos et al., 2019; John et al., 2014a, 2012; Zucoloto, Maroco & Campos, 2016) and our observed measurement invariance provide support to the external validity of the results for the broader population.

Despite these limitations, it is expected that the results presented will support use of an instrument to evaluate perception of OA in different contexts using a simple and standardized measurement method. This may contribute to future research and clinical practice as it may contribute in elaborating a clinical treatment plan that is consistent with patient expectations.

## Conclusions

Scores of the OES-Pt were valid and reliable in Brazilian adult individuals. Age, sex and SES were weak or not statistically significantly related to OA.

## Supplemental Information

10.7717/peerj.8814/supp-1 Supplemental Information 1 Raw Data.

## Additional Information and Declarations

### Data Availability

The following information was supplied regarding data availability: The raw measurements are available as a Supplemental File.
